# Underestimation of the Maximal Capacity of the Mitochondrial Electron Transport System in Oligomycin-Treated Cells

**DOI:** 10.1371/journal.pone.0150967

**Published:** 2016-03-07

**Authors:** Juliana S. Ruas, Edilene S. Siqueira-Santos, Ignacio Amigo, Erika Rodrigues-Silva, Alicia J. Kowaltowski, Roger F. Castilho

**Affiliations:** 1 Departamento de Patologia Clínica, Faculdade de Ciências Médicas, Universidade Estadual de Campinas (UNICAMP), Campinas, SP, Brazil; 2 Departamento de Bioquímica, Instituto de Química, Universidade de São Paulo (USP), São Paulo, SP, Brazil; Duke University Medical Center, UNITED STATES

## Abstract

The maximal capacity of the mitochondrial electron transport system (ETS) in intact cells is frequently estimated by promoting protonophore-induced maximal oxygen consumption preceded by inhibition of oxidative phosphorylation by oligomycin. In the present study, human glioma (T98G and U-87MG) and prostate cancer (PC-3) cells were titrated with different concentrations of the protonophore CCCP to induce maximal oxygen consumption rate (OCR) within respirometers in a conventional growth medium. The results demonstrate that the presence of oligomycin or its A-isomer leads to underestimation of maximal ETS capacity. In the presence of oligomycin, the spare respiratory capacity (SRC), i.e., the difference between the maximal and basal cellular OCR, was underestimated by 25 to 45%. The inhibitory effect of oligomycin on SRC was more pronounced in T98G cells and was observed in both suspended and attached cells. Underestimation of SRC also occurred when oxidative phosphorylation was fully inhibited by the ATP synthase inhibitor citreoviridin. Further experiments indicated that oligomycin cannot be replaced by the adenine nucleotide translocase inhibitors bongkrekic acid or carboxyatractyloside because, although these compounds have effects in permeabilized cells, they do not inhibit oxidative phosphorylation in intact cells. We replaced CCCP by FCCP, another potent protonophore and similar results were observed. Lower maximal OCR and SRC values were obtained with the weaker protonophore 2,4-dinitrophenol, and these parameters were not affected by the presence of oligomycin. In permeabilized cells or isolated brain mitochondria incubated with respiratory substrates, only a minor inhibitory effect of oligomycin on CCCP-induced maximal OCR was observed. We conclude that unless a previously validated protocol is employed, maximal ETS capacity in intact cells should be estimated without oligomycin. The inhibitory effect of an ATP synthase blocker on potent protonophore-induced maximal OCR may be associated with impaired metabolism of mitochondrial respiratory substrates.

## Introduction

Oxygen consumption rate (OCR) measurements are one of the preferred methods for mitochondrial function or dysfunction evaluation in cultured cells (for reviews see [1], [2]). When the plasma membrane is permeabilized or isolated mitochondria are studied, the respiratory control ratio can be assessed in a medium supplemented with respiratory substrates by measuring the increase in OCR after the addition of ADP. When using intact cells, specific respirometric protocols are usually employed to evaluate mitochondrial function and can provide valuable information such as that described below. The fraction of basal OCR (or ROUTINE respiration, as it is referred to in [2]) inhibited by addition of the ATP synthase inhibitor oligomycin gives an estimate of the respiration rate necessary to sustain cellular ATP turnover under basal conditions. The respiration remaining in the presence of oligomycin is linked to the proton leak rate across the mitochondrial membrane and to other processes such as reactive oxygen species formation and energy-driven ion/metabolite transport. Maximal capacity of the mitochondrial electron transport system (ETS) can be estimated by inducing maximal OCR via chemical dissipation of the mitochondrial membrane potential, generally by the addition of a potent protonophore such as CCCP or FCCP. However, assessment of this maximal OCR usually requires caution (to avoid underestimating the results and drawing incorrect conclusions) as well as titration of the protonophore [1, 2]. The presence of oligomycin during the estimation of maximal OCR is widespread in such assays and it seems to be important to prevent the reverse activity of ATP synthase with rapid intracellular ATP depletion, which may lead to cellular metabolic dysfunction and death. Spare respiratory capacity (SRC) is given by the difference between maximal OCR and basal respiration and is an estimative of the cell’s ability to cope with large increases in ATP turnover. Finally, the addition of a potent respiratory chain inhibitor, such as antimycin A, allows non-mitochondrial OCR to be estimated.

Mitochondrial energy metabolism seems to play specific roles in the biology of tumor cells [3–6]. Although mutations in citric acid cycle enzymes are associated with tumor formation [7, 8], most tumor cells present normal mitochondrial integrity and oxidative phosphorylation capacity [9–11]. Recently, two groups showed that mitochondrial respiration is essential for tumor cell proliferation since it promotes aspartate biosynthesis [12, 13]. Further assessment of mitochondrial function in tumor cells may contribute to a better understanding of the role of these organelles in tumorigenesis and to the development of effective new cancer therapies [11, 14, 15]. In this study we investigated the effect of ATP synthase inhibitors on maximum OCR measured in tumor cells in order to understand our experimental evidences of an undesirable inhibitory effect of the ATP synthase inhibitor oligomycin on maximal OCR obtained in studies evaluating mitochondrial function in glioma cell lines. The data presented here indicate that the presence of oligomycin significantly underestimates CCCP- or FCCP-induced maximal OCR and SRC. Moreover, alternative compounds and protocols to assess maximal OCR in cells with inhibited oxidative phosphorylation were evaluated.

## Materials and Methods

### Chemicals and Cell Lines

Most of the chemicals used, including adenosine diphosphate (ADP; A2754), bongkrekic acid (BKA; B6179), carbonyl cyanide 3-chlorophenylhydrazone (CCCP; C2759), carboxyatractyloside (CAT; C4992), carbonyl cyanide 4-(trifluoromethoxy)phenylhydrazone (FCCP; C2920), digitonin (D141), dimethyl sulfoxide (DMSO; D8418), 2,4-dinitrophenol (DNP; D198501), 4-(2-hydroxyethyl)piperazine-1-ethanesulfonic acid (HEPES; H3375), oligomycin (oligo; O4876), oligomycin A (oligo A; 75351) and sodium pyruvate (P4562) were obtained from Sigma-Aldrich (St Louis, MO, USA). Oligomycin (oligo*; item number 11341) was also obtained from Cayman Chemical Company (Ann Arbor, MI, USA), as was citreoviridin (citre; item number 11319). CCCP, citreoviridin, DNP, FCCP, oligomycin and oligomycin A stock solutions were prepared by dissolving the respective chemicals in DMSO. ADP and HEPES solutions were prepared in ultrapure (Type I) water, and their pH adjusted to 7.2 with NaOH. Carboxyatractyloside was dissolved in Type I water. The cocktail of NADH-linked respiratory substrates containing glutamate, α-ketoglutarate, malate and pyruvate was prepared by dissolving the respective acids in water and adjusting the pH to 7.2 with KOH.

Cell lines T98G and U-87MG were derived from human glioblastoma, while PC-3 was derived from a prostate cancer bone metastasis. All three cell lines were obtained from ATCC (Manassas, VA, USA). Antibiotics (penicillin and streptomycin); Dulbecco’s modified Eagle’s medium (DMEM), containing 2 g/L of glucose; fetal bovine serum (FBS); phosphate buffered saline (PBS); and trypsin/EDTA solution (0.25%) were purchased from Vitrocell (Campinas, SP, Brazil).

### Cell Cultures

PC-3, T98G and U-87MG cells were maintained continuously at 37°C in a humidified 5% CO_2_ and 95% air atmosphere in 175 cm^2^ tissue culture flasks and DMEM supplemented with 10% FBS and antibiotics (100 U/mL penicillin and 100 μg/mL streptomycin). Because the doubling times of these cells are between 24 and 36 h, the cells were seeded every two or three days (approximately 25,000 cells/cm^2^) into new culture flasks and fresh medium. Briefly, the adhered cells were pre-washed with PBS and then incubated for 2–4 min with a trypsin/EDTA solution (0.25%) at 37°C to dissociate the cells from the flask surface. Trypsin was inactivated by adding supplemented DMEM to the flask. Passage of the cells into new flasks and fresh medium was performed after centrifuging the cell suspension at 400 *g* for 5 min and suspending the resulting pellet in supplemented DMEM. The experiments were conducted when the cells were between the 3^rd^ and 16^th^ passages.

For experiments with suspended cells, these were plated on 150 cm^2^ culture dishes at an initial density of 15–20,000 cells/cm^2^ and maintained for 3 or 4 days. On the day of the experiments, trypsin-dissociated cells were centrifuged at 400 *g* for 5 min and suspended in DMEM containing 20 mM HEPES-Na^+^ at a density of 24–32 x 10^6^/mL (>95% viability). Cell suspensions were kept at room temperature and used within 2.5 hours.

For the experiments with attached cells, T98G cells were seeded in Seahorse XF24 V7 cell culture microplates at a density of 40–50,000 cells/well in supplemented DMEM. After 24 h, the medium was replaced by supplemented DMEM containing 20 mM HEPES-Na^+^ and left for 1 h in a CO_2_ incubator to allow the cells to stabilize before the experiment.

### Isolation of rat forebrain mitochondria

Male Wistar rats (*Rattus norvegicus albinus*) were obtained from the State University of Campinas (UNICAMP) Animal Breeding Center. Six rats were used for the present study and were euthanized by decapitation [16]. Rat brain mitochondria were isolated as described by Michelini et al. [17] using digitonin to permeabilize the synaptosomal plasma membrane [18]. The use of a similar procedure to isolate mitochondria from intact cultured glioma cells did not result in functional mitochondria in sufficient quantity to conduct experiments (data not shown). All procedures involving animal handling were approved by the Ethics Committee of Universidade Estadual de Campinas (protocol number CEUA 2534–1) and are in accordance with the Brazilian National Guide.

### Oxygen uptake measurements in suspended tumor cells and isolated rat brain mitochondria

The OCR in suspended intact cells and isolated rat brain mitochondria was measured using a high-resolution respirometer (OROBOROS Oxygraph-2k, Innsbruck, Austria) with DatLab 4 software for data acquisition and analysis. In intact tumor cells it was measured by incubating the cells (3–4 x 10^6^) at 37°C in a 2 mL chamber containing supplemented DMEM and 20 mM HEPES-Na^+^. The pH of the medium at the beginning of the experiments was between 7.30 and 7.45.

The OCR in permeabilized cells and isolated rat brain mitochondria was measured by incubating the cells (3–4 x 10^6^) or mitochondria (0.6 mg) at 37°C in a 2 mL chamber containing 125 mM sucrose, 65 mM KCl, 10 mM HEPES-K^+^ pH 7.2, 2 mM K_2_HPO_4_, 1 mM MgCl_2_, 1 mM EGTA and a cocktail of respiratory substrates (α-ketoglutarate, malate, glutamate and pyruvate; 5 mM of each). Digitonin (30 μM) was also present in the reaction medium for the experiments with permeabilized cells.

Before each experiment, the oxygen concentration in the medium was equilibrated for 2–3 min with air in the respirometer chambers at 37°C until a stable signal was obtained at an oxygen concentration of approximately 195 μM. Cell suspensions or isolated rat brain mitochondria were then added to a final volume of 2 mL, and the chambers were closed by inserting the stoppers. The cell suspensions were constantly stirred (750 rpm) inside the chambers, and the chemicals were added through the titanium injection port of the stoppers using Hamilton syringes. All measurements were carried out when oxygen concentration was above 30 μM in the reaction medium. OCR was calculated as the negative time derivative of oxygen concentration measured in the closed respirometer chambers and expressed per million of cells or per mg of isolated mitochondria. Data were recorded at 2 s intervals, and 10 data points were used to calculate the slope of the OCR plot through a polynomial fit with DatLab 4 software [2].

### Oxygen uptake measurements in attached T98G cells

Oxygen consumption in attached T98G cells was measured using a Seahorse XF24 Analyzer. Initially, 300 μL of supplemented DMEM containing 20 mM HEPES-Na^+^ was placed in each well, and each addition consisted of 75 μL, resulting in a final volume of 600 μL. The first injector was loaded with either medium plus oligomycin or medium containing the corresponding volume of DMSO. After the first addition, the oligomycin concentration in the wells was 1 μg/mL. The second injector was loaded with CCCP to achieve a concentration of 4 μM in the wells, while the other two injectors were loaded with CCCP to achieve concentrations of 5.4 and 6.8 μM. Four time points were measured before the first injection (basal respiration), three after the oligomycin/DMSO addition and two after each CCCP addition. At the end of each experiment, the cells were washed twice with PBS and their total protein content determined by the Bradford method in each well to normalize the OCR values. Data points for each condition were pooled and calculated as the percentage change from basal respiration.

### Statistical analysis

Results are presented as representative traces or as means ± standard errors (SEM). Each experimental protocol was conducted at least in duplicate, with a minimum of four different cell culture passages or mitochondrial preparations. Since the measurements were always made with matched samples, i.e., control and treated conditions, two-tailed paired Student's t-test was used to compare two different groups. Repeated-measures one-way ANOVA followed by the post hoc Bonferroni test was used when multiple comparisons were performed. Differences between the concentrations of CCCP and FCCP to reach maximal OCR were assessed by the Mann-Whitney test. The level of significance was set at *P*<0.05. The data were analyzed using GraphPad Prism 5 software.

## Results

### The inhibitory effect of oligomycin on CCCP-induced maximal OCR in suspended tumor cells

**Fig 1:**
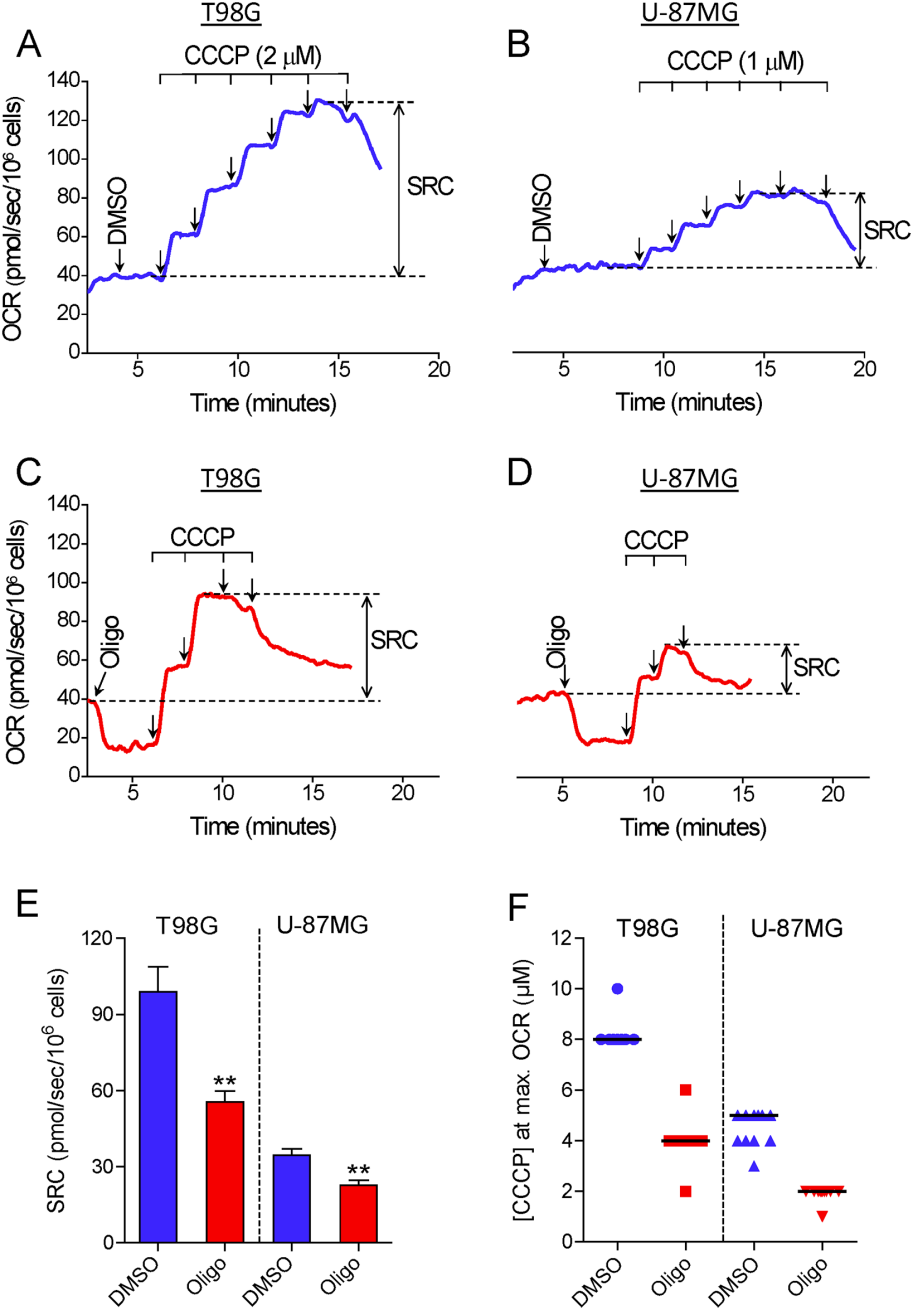
Inhibitory effect of oligomycin on CCCP-induced maximal oxygen consumption in intact human glioma cells. **A** and **C**: Representative oxygen consumption rate (OCR) traces in suspended T98G cells (1.5×10^6^/mL). Where indicated by the arrows, 1 μg/mL oligomycin (Oligo) and DMSO (0.5 μL of each) were added, followed by sequential additions of CCCP (2 μM each). **B** and **D**: Representative traces of OCR in suspended U-87MG cells (2.0×10^6^ cells/mL). Where indicated by the arrows, 1 μg/mL oligomycin and 0.5 μL DMSO were added followed by sequential additions of CCCP (1 μM each). SRC was estimated as the difference between maximal and basal OCR, as shown in the representative traces. **E**: SRC values for T98G and U-87MG cells obtained in the presence and absence of oligomycin. **Statistically significant difference from the result for the respective control (DMSO), *P*<0.01. **F**: Values of CCCP concentrations required to achieve maximal OCR in T98G and U-87MG cells in the presence and absence of oligomycin.

Fig 1 shows the results of experiments conducted to determine the maximal OCR in suspended intact glioma cells. T98G and U-87MG cells were incubated in HEPES-buffered, FBS-supplemented (10%) DMEM. This medium contains 11 mM glucose, 4 mM glutamine and 1.25 mM pyruvate as major oxidative substrates. The purpose in choosing this incubation medium was to simulate similar conditions to those in the cell culture. First, maximal OCR was determined by titration with the protonophore CCCP. In most of the experiments, T98G and U-87MG cells reached maximal OCR in the presence of 8 and 4–5 μM CCCP, respectively (Fig 1A, 1B and 1F). Remarkably, when a standard experimental protocol to determine maximal OCR consisting of CCCP titration preceded by oligomycin-inhibited oxidative phosphorylation was used, a lower maximal OCR was observed for both glioma cells (Fig 1C and 1D). In most of the experiments with T98G and U-87MG cells previously treated with oligomycin, maximal OCR was obtained after the addition of 4 and 2 μM CCCP, respectively (Fig 1F). SRC, i.e., the difference between maximal and basal OCR, was underestimated by 42.8 ± 2.7% and 34.3 ± 2.8% in oligomycin-treated T98G and U-87MG cells, respectively, compared with cells treated only with the DMSO vehicle before CCCP titration (Fig 1E). As previously reported for isolated mitochondria and intact cells [2, 19–21], inhibition of OCR was observed in our experiments when supraoptimal concentrations of CCCP were present. This undesired effect of high concentrations of CCCP on OCR may be related to an excess of this compound in the mitochondrial membrane, leading to inhibition of respiratory activity [19], and/or due to a decline in the supply of respiratory substrates to mitochondria [1]. The concentrations of CCCP necessary to reach maximal OCR varied during our study. This may be related to the binding of CCCP to components of the different batches of FBS. In addition, we observed that this variation was minimized by avoiding repeated freezing and thawing of the aliquots of CCCP solution. Despite this variation, the concentration of CCCP required to reach maximal OCR in oligomycin-treated cells was always approximately half that required when only DMSO (the oligomycin solvent) was present.

To evaluate whether the inhibitory effect of oligomycin on CCCP-induced maximal OCR would also be observed with a different cell line, we conducted experiments with PC-3 cells, derived from prostatic adenocarcinoma. S1 File shows that compared with the value for PC-3 cells treated with DMSO, SRC was underestimated by 26.3 ± 3.6% in PC-3 cells treated with oligomycin.

Most of the next experiments used T98G cells, since they showed greater sensitivity to the inhibitory effect of oligomycin on CCCP-induced maximal OCR. Experiments were conducted to determine whether the inhibitory effect of oligomycin on CCCP-induced maximal OCR would be observed with a single addition of the optimal concentration of CCCP (S2 File). CCCP titration or a single addition of the optimal concentration of CCCP resulted in similar maximal OCRs for T98G cells (S2A and S2B File). However, when the optimal concentration of CCCP was added to T98G cells previously treated with oligomycin, a lower maximal OCR was observed and was followed by progressive inhibition of oxygen consumption (S2C File).

**Fig 2:**
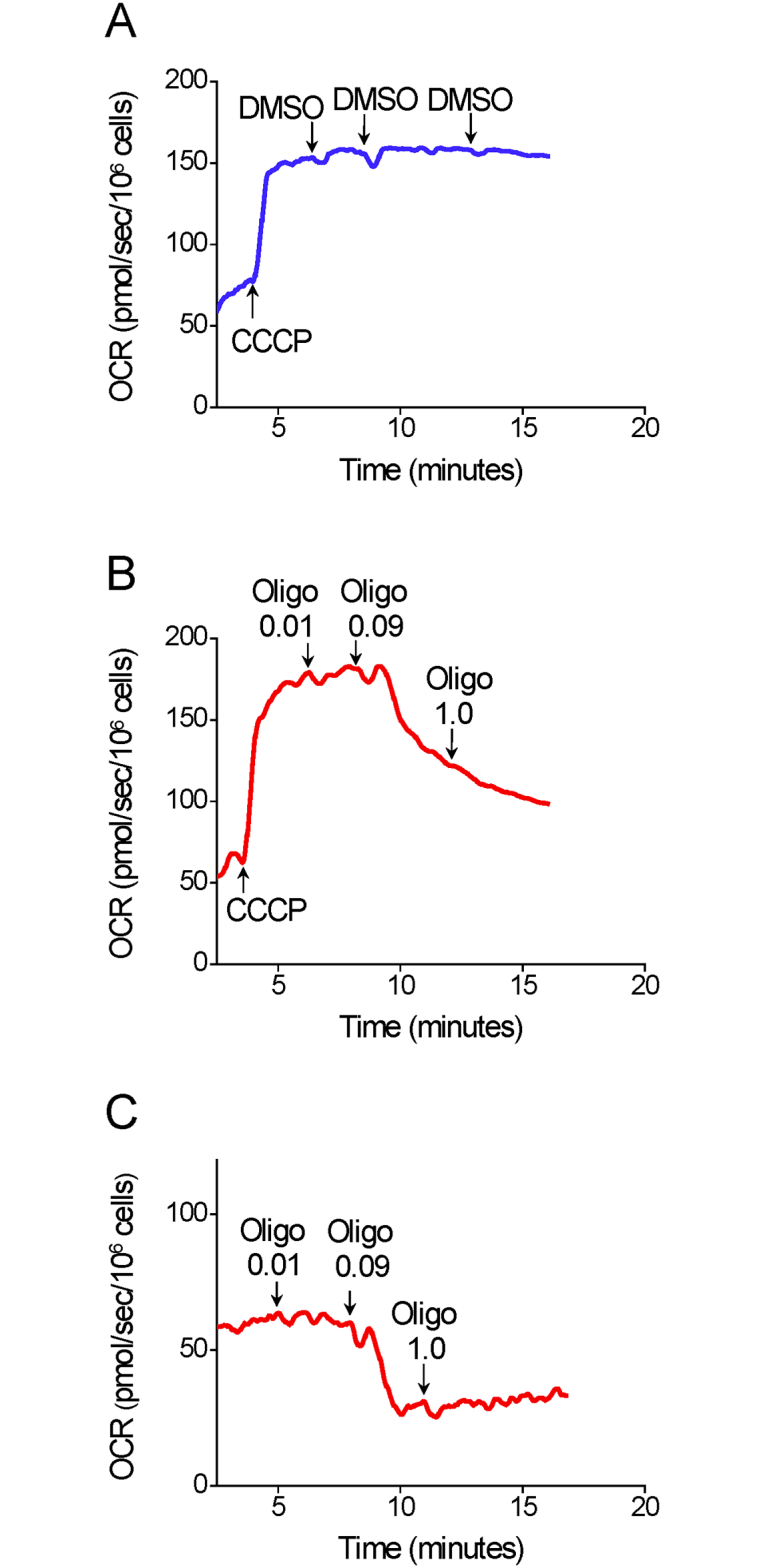
Inhibitory effect of oligomicyn addition on CCCP-induced maximal OCR in T98G cells. **A–C**: Representative OCR traces in suspended T98G cells (1.5×10^6^ cells/mL). Where indicated by the arrows, 5 μM CCCP and 0.5 μL of oligomycin (Oligo; 0.01, 0.09 and 1.0 μg/mL) or DMSO were added.

We also investigated whether the addition of oligomycin would have an inhibitory effect on the OCR of cells already stimulated by CCCP. A suboptimal concentration of CCCP was used to avoid any delayed inhibitory effect of this compound on OCR during the experiment (Fig 2A). The results indicated that the addition of oligomycin at a concentration high enough to inhibit oxygen consumption by oxidative phosphorylation, i.e., 0.1 μg/mL (Fig 2C), results in remarkable inhibition of CCCP-stimulated OCR for T98G cells (Fig 2B).

### The inhibitory effect of oligomycin on CCCP-induced maximal OCR in attached T98G cells

**Fig 3:**
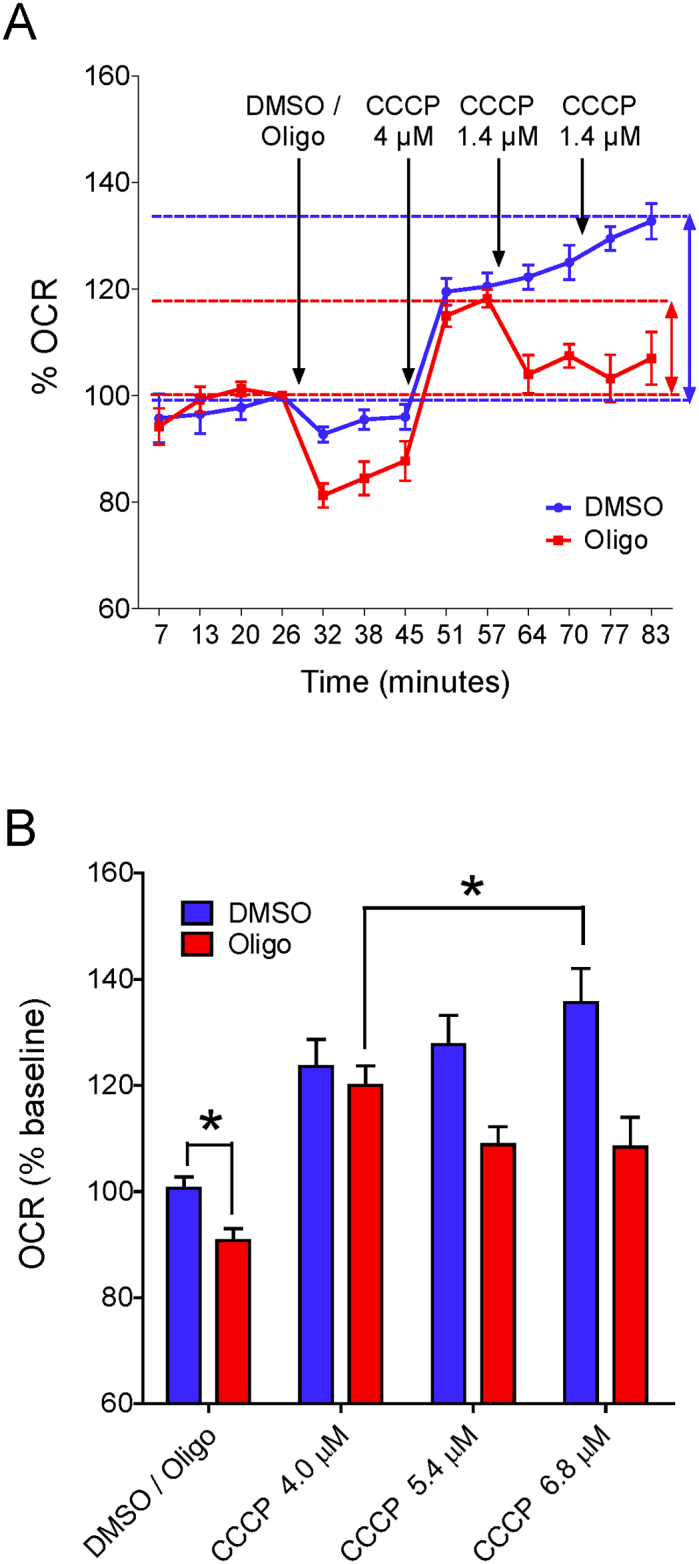
Inhibitory effect of oligomycin on CCCP-induced maximal OCR in attached T98G cells. **A**: Representative experiment to determine OCR in attached T98G cells. Arrows indicate additions of reagents and their concentrations. Results are shown as percentages in relation to the last point before the first addition. SRC (i.e., the difference between maximal respiratory rate and basal respiration) for each condition is indicated by vertical coloured arrows. **B**: Quantification of OCR with respect to basal respiration in cells treated with DMSO or oligomycin. Maximal respiratory rates with CCCP were smaller in oligomycin-treated cells and occurred at lower CCCP concentrations. *Statistically significant difference from the result for DMSO, *P* < 0.05.

Fig 3 shows measurements of OCR in attached T98G cells. Maximal OCR was achieved by adding CCCP three consecutive times to give final protonophore concentrations of 4, 5.4 and 6.8 μM, respectively. Higher CCCP concentrations, e.g., 7.1 or 8.3 μM were not used because pilot experiments indicated that these did not result in further increases in OCR (results not shown). Like suspended T98G cells, attached T98G cells showed lower OCR and SRC when CCCP titration was preceded by addition of oligomycin (Fig 3A). In this case, maximal OCR was reached with 4 μM CCCP, while when only DMSO was added the corresponding figure was 6.8 μM CCCP (Fig 3B). Compared with the value for T98G cells treated with DMSO, SRC was underestimated by 43.7 ± 2.3% in T98G cells treated with oligomycin (Fig 3B).

### Evaluation of alternative protocols to estimate maximal OCR in intact tumor cells

**Fig 4:**
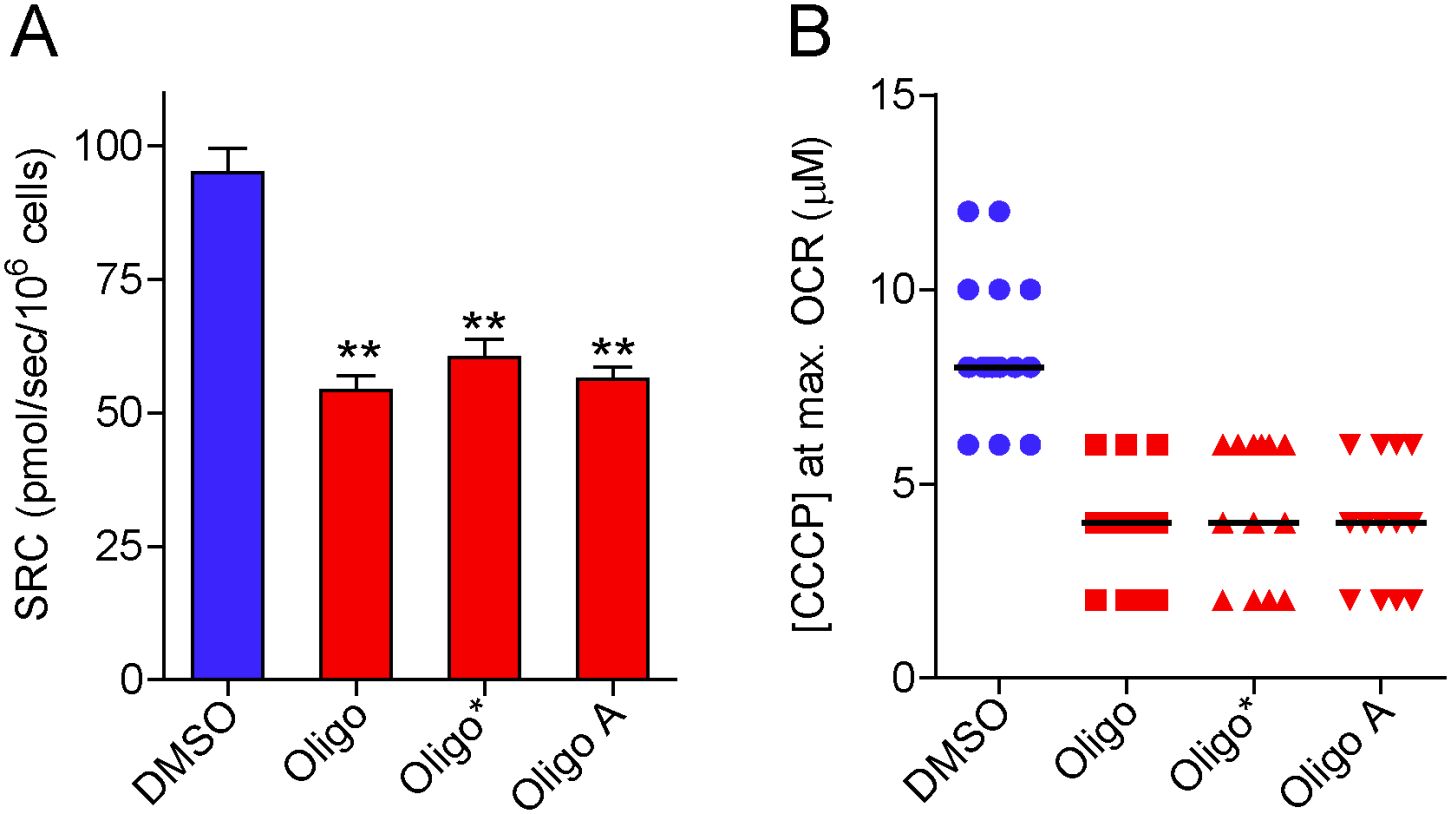
Inhibitory effect of oligomycin and its A-isomer on SRC for T98G cells. After incubation of T98G cells (1.5×10^6^ cells/mL) for 3–5 min, 1 μg/mL oligomycin from Sigma-Aldrich (Oligo), 1 μg/mL oligomycin from Cayman Chemical (Oligo*), 1 μg/mL oligomycin A (Oligo A) or 0.5 μL DMSO were added, followed by sequential additions of CCCP (2 μM each). **A**: SRC values for suspended T98G cells in the absence and presence of oligomycin or its A-isomer. **Statistically significant difference from the result for DMSO, *P*<0.01. **B**: Values of CCCP concentrations required to achieve maximal OCR in T98G cells in the presence and absence of oligomycin or its A-isomer.

In view of the preceding results indicating that oligomycin has an important inhibitory effect on CCCP-induced maximal OCR in intact tumor cells, the next experiments were conducted to test different experimental protocols for maximal OCR assessment in T98G with inhibited oxidative phosphorylation. First, oligomycin from a different supplier (referred to here as oligo*) was tested. The effect of the A-isomer of oligomycin (oligo A) was also evaluated (Fig 4). The oligomycin complex is a mixture of oligomycins A, B and C, the first two being the most potent ATP synthase inhibitors [22]. The results in Fig 4 show that oligo* or oligo A had an inhibitory effect on SRC in T98G cells similar to that observed with oligomycin.

**Fig 5:**
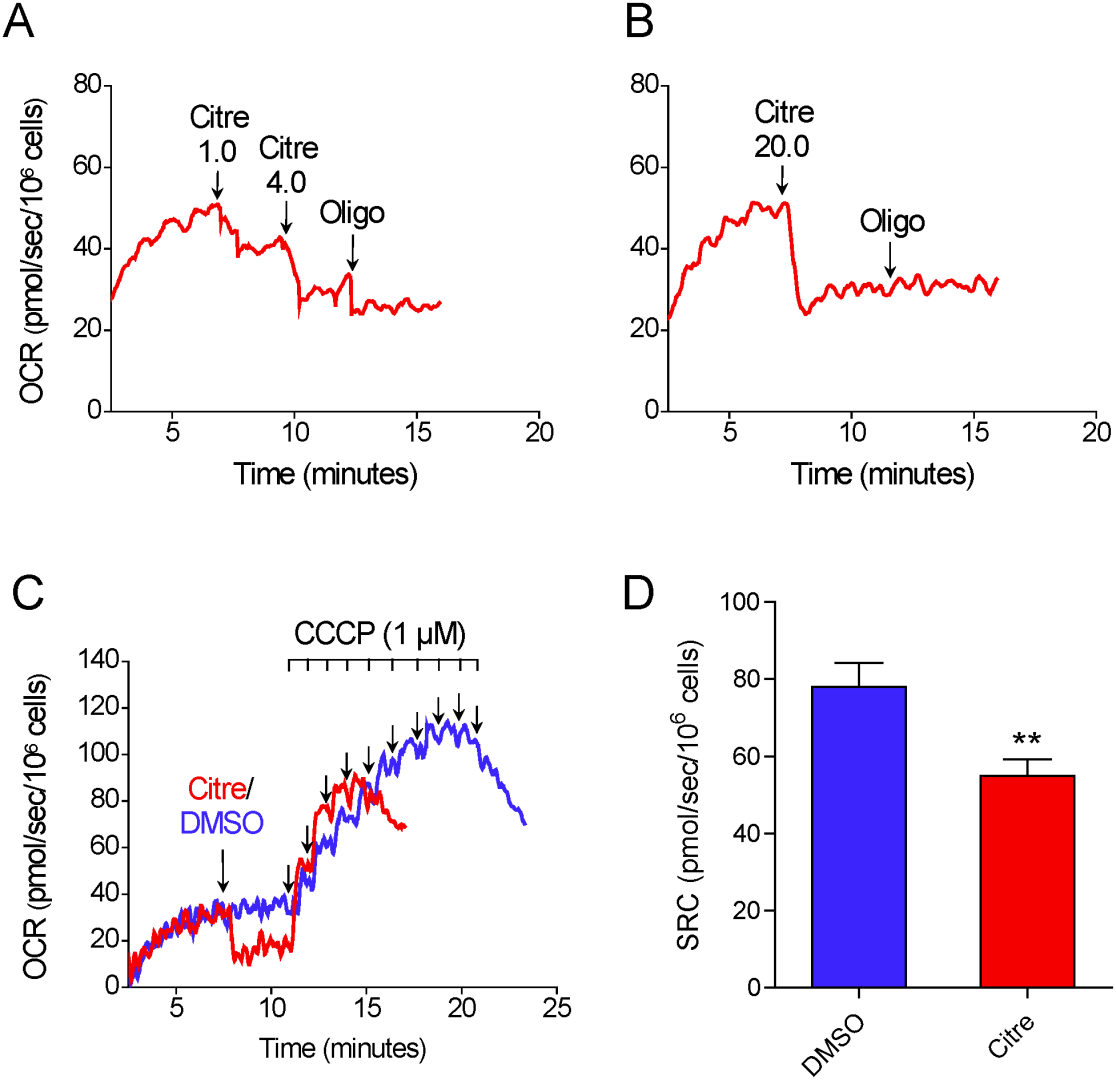
Inhibitory effect of citreoviridin on CCCP-induced maximal oxygen consumption in T98G cells. **A** and **B**: Representative OCR traces in suspended T98G cells (1.5×10^6^ cells/mL). Where indicated by the arrows, citreoviridin (Citre; 1 μM, 4 μM and 20 μM) and 1 μg/mL oligomycin (Oligo) were added. **C**: Representative traces of OCR in T98G cells. Where indicated by the arrows, 1 μL DMSO or 20 μM citreoviridin were added followed by sequential additions of CCCP (1 μM each). **D**: SRC values for T98G cells in the presence and absence of 20 μM citreoviridin. **Statistically significant difference from the results for DMSO, *P*<0.01.

The effect of the ATP synthase inhibitor citreoviridin was also tested on CCCP-induced maximal OCR in T98G cells. Unlike oligomycin, which binds to the F_O_ subunit of ATP synthase [23, 24], citreoviridin targets the F_1_ subunit [24, 25]. Fig 5A and 5B) shows that the addition of 5 μM citreoviridin partially inhibits oxygen consumption under basal conditions, indicating inhibition of oxidative phosphorylation. Nevertheless, sequential addition of oligomycin still resulted in further inhibition of oxygen consumption (Fig 5A). When a higher concentration of citreoviridin was used (20 μM), no further inhibitory effect was observed for oligomycin (Fig 5B). This 20 μM concentration of citreoviridin was used for the next experiments. The results in Fig 5C and 5D) show that, as with oligomycin (but to a lesser extent), when oxidative phosphorylation was fully inhibited by citreoviridin, CCCP-induced maximal OCR was underestimated. In the presence of citreoviridin, SRC was underestimated by 26.4 ± 6.5% compared with DMSO-treated T98G cells.

**Fig 6:**
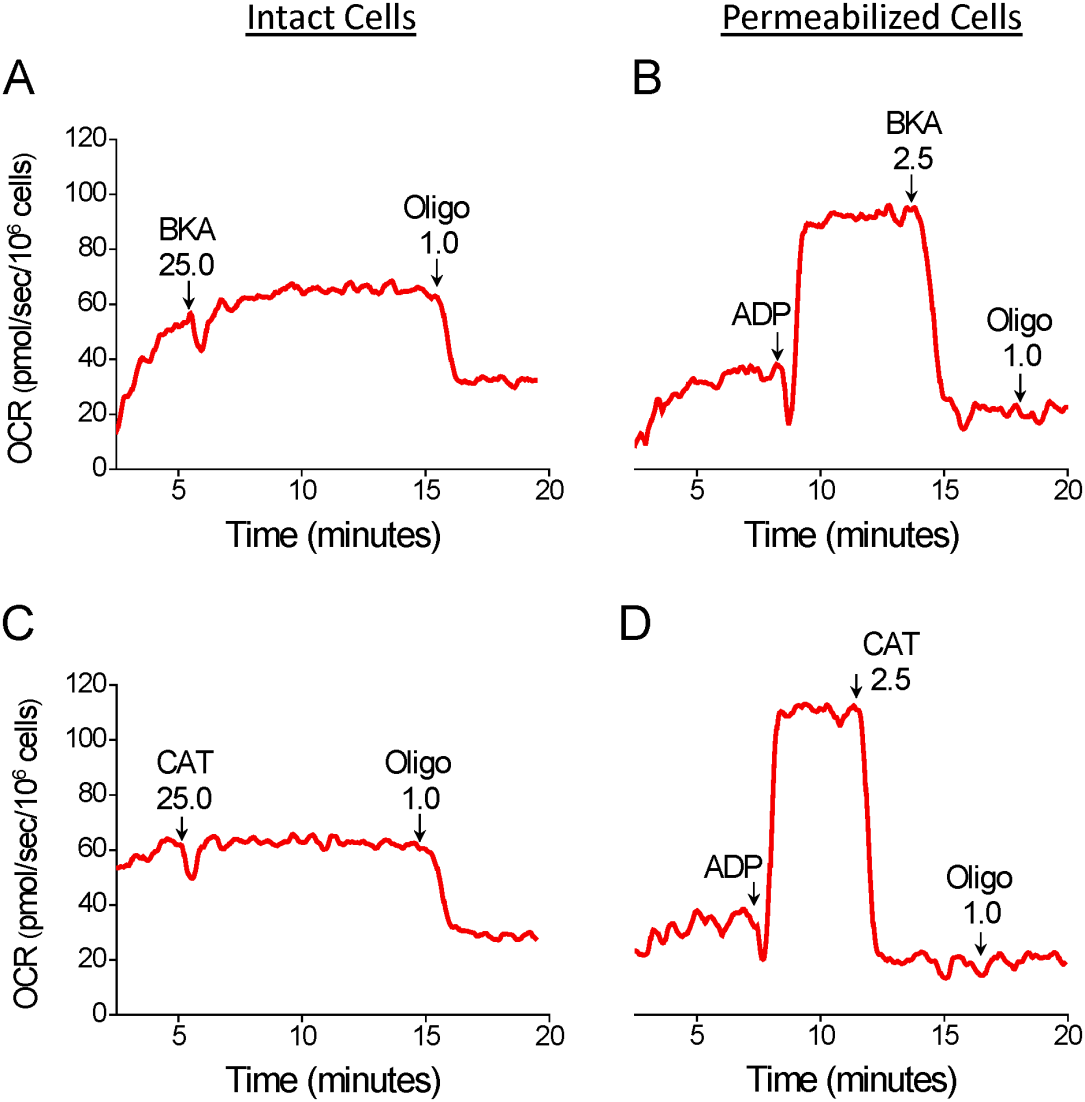
Effect of bongkrekic acid (BKA) and carboxyatractyloside (CAT) on oxygen consumption due to oxidative phosphorylation in intact and digitonin-permeabilized T98G cells. **A** and **C**: Representative OCR traces in intact T98G cells. Suspended T98G cells (1.5×10^6^ cells/mL) were incubated in the medium for intact cells and, where indicated by the arrows, 25 μM BKA, 25 μM CAT and 1 μg/mL oligomycin (Oligo) were added. **B** and **D**: Representative traces of OCR in digitonin-permeabilized T98G cells. A 125-μL aliquot of T98G (3×10^6^ cells) cell suspension was added to the final volume of 2 mL of reaction medium (125 mM sucrose, 65 mM KCl, 10 mM HEPES-K^+^ pH 7.2, 2 mM K_2_HPO_4_, 1 mM MgCl_2_, 1 mM EGTA and a cocktail of the respiratory substrates α-ketoglutarate, malate, glutamate and pyruvate, 5 mM of each) containing 30 μM digitonin. Where indicated by the arrows, 800 μM ADP, 2.5 μM BKA, 2.5 μM CAT and 1 μg/mL oligomycin (Oligo) were added.

A different approach for the inhibition of oxidative phosphorylation in intact T98G cells was tested by using the adenine nucleotide translocator (ANT) inhibitors bongkrekic acid (BKA) and carboxyatractyloside (CAT) [26, 27]. Fig 6 shows the results with intact and permeabilized T98G cells. The additions of BKA or CAT (25 μM of each, **A**, **C**) to intact cells had no effect on basal oxygen consumption, indicating that these compounds do not inhibit oxidative phosphorylation in intact glioma cells. As expected, the sequential addition of oligomycin resulted in significant inhibition of oxygen consumption. Experiments with digitonin-permeabilized T98G cells indicated that BKA and CAT (2.5 μM of each, **B**, **D**) were effective in fully inhibiting ADP-stimulated oxygen consumption.

Next, we tested whether similar results would also be observed when CCCP is replaced by another potent protonophore or by a weaker protonophore, respectively FCCP or 2,4-dinitrophenol (DNP); the latter is less potent than CCCP and FCCP by two orders of magnitude [28]. The results when CCCP was replaced by FCCP were similar to those when CCCP was used (S3 File). Compared with the values for T98G cells treated with the vehicle DMSO before FCCP titration, SRC was underestimated by 36.7 ± 1.8% in T98G cells treated with oligomycin (S3C File). As expected, the concentrations of FCCP (median concentration of 5 μM) required to achieve maximal OCR in T98G cells were lower (*P*<0.01) (S3D File) than those required when CCCP was used (median concentration of 8 μM).

**Fig 7:**
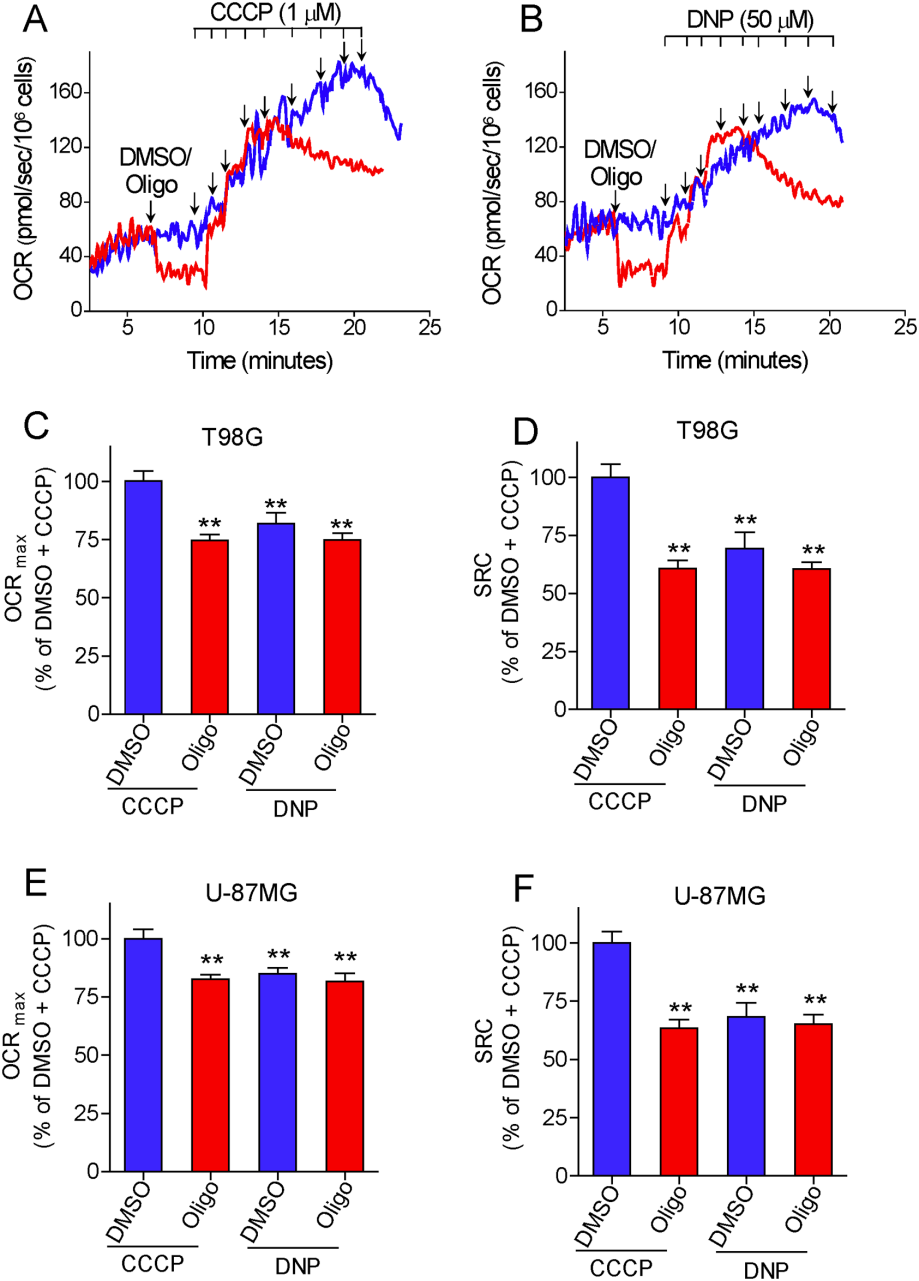
Effect of oligomycin on 2,4-dinitrophenol (DNP)-induced maximal oxygen consumption in intact human glioma cells. **A** and **B**: Representative oxygen consumption rate (OCR) traces of suspended T98G cells (1.5×10^6^/mL). Where indicated by the arrows, 1 μg/mL oligomycin (Oligo) and DMSO (0.5 μL of each) were added, followed by sequential additions of CCCP (1 μM each) (**A**) or DNP (50 μM each) (**B**). **C** and **E**: Maximal OCR (OCR_max_) values for T98G (**C**) and U-87MG cells (**E**) determined by sequential additions of CCCP or DNP, in the presence and absence of oligomycin. Data are expressed as percentage of OCR_max_ obtained with CCCP titration in DMSO-treated cells (% of DMSO + CCCP). **Statistically significant difference from the results for the “DMSO + CCCP” group, *P*<0.01. **D** and **F**: SRC values for T98G (**D**) and U-87MG cells (**F**) determined by sequential additions of CCCP or DNP, in the presence and absence of oligomycin. Data are expressed as percentage of SRC obtained with CCCP titration in DMSO-treated cells (% of DMSO + CCCP). **Statistically significant difference from the results for the “DMSO + CCCP” group, *P*<0.01.

Interestingly, when CCCP was replaced by DNP, lower maximal OCRs were observed for T98G and U-87MG cells (Fig 7, 7B, 7C and 7E), reaching similar values to those obtained with CCCP titration when oligomycin is present. The median DNP concentrations required for maximal OCR were 350 μM and 200 μM for T98G cells in the absence and presence of oligomycin and 250 μM and 150 μM for U-87MG cells in the absence and presence of oligomycin, respectively. The presence of oligomycin did not significantly decrease the SRC estimated by DNP titration, but the SRC values for both cells were similar to those obtained by CCCP titration in oligomycin-treated cells.

**Fig 8:**
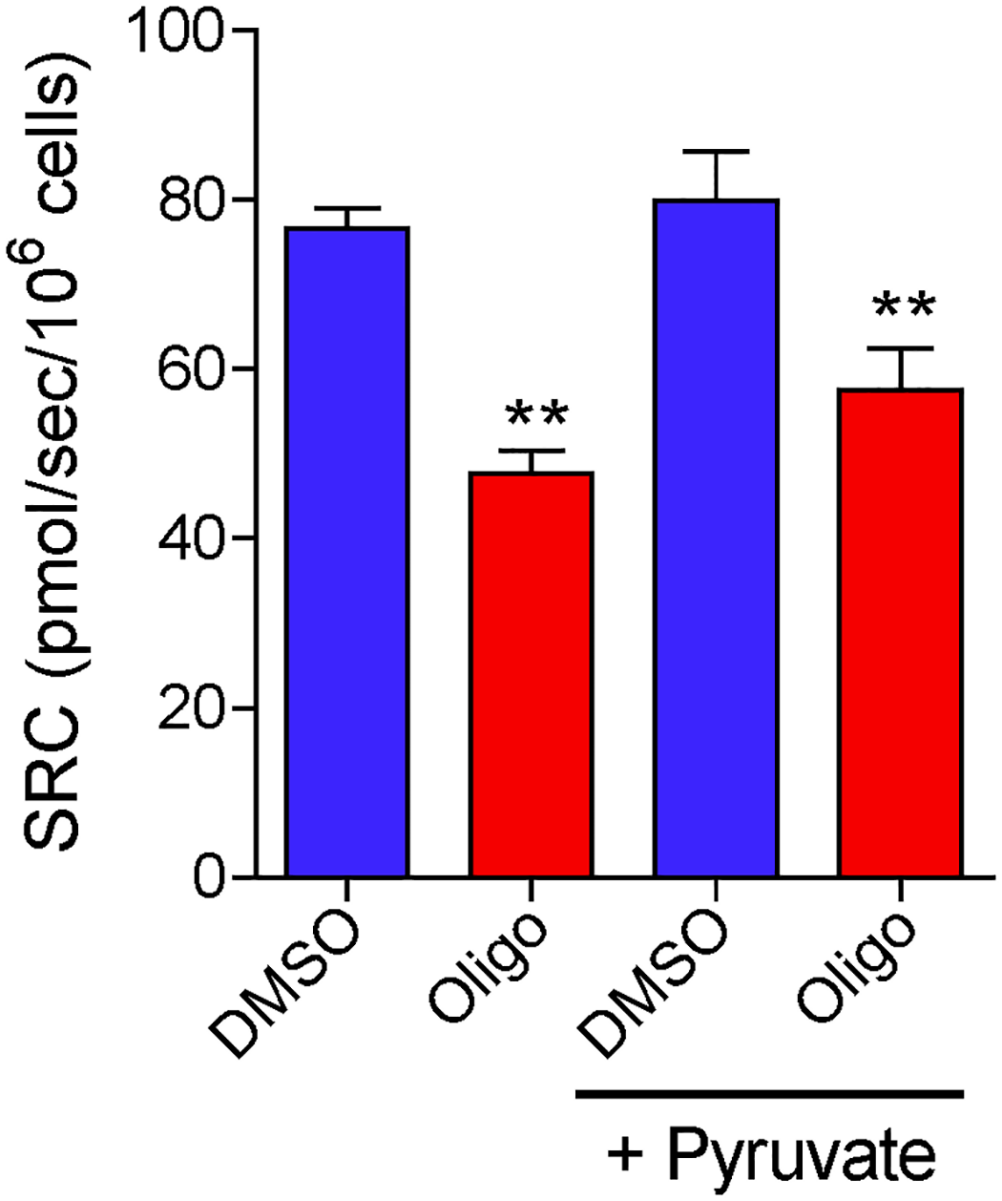
Pyruvate supplementation does not prevent the inhibitory effect of oligomycin on SRC determined for T98G cells. T98G cells (1.5×10^6^ cells/mL) were incubated in the presence or absence of oligomycin (Oligo; 1 μg/mL). Where indicated, the incubation medium was supplemented with 10 mM sodium pyruvate (+ Pyruvate). Maximal OCR for suspenced T98G cells was determined by CCCP titration and SRC was calculated. **Statistically significant difference from the results for DMSO, *P*<0.01.

The absence of pyruvate in the incubation medium can underestimate the maximal OCR induced by FCCP in some cell lines and synaptosomes [29, 30]. We thus tested if pyruvate supplementation could eliminate the effects of oligomycin inhibiting maximal OCR obtained with protonophore (CCCP). Our results (Fig 8) indicate that pyruvate supplementation (10 mM) does not prevent the inhibitory effect of oligomycin on SRC determined for T98G cells. In addition, no significant difference was observed in basal and maximal OCR with pyruvate supplementation (results not shown). The lack of effect of pyruvate under our conditions may be due to the weak mitochondrial oxidation of this substrate in high glycolytic tumor cells, which can use glutamine as a preferential respiratory substrate [31, 32].

### Oligomycin has little or no inhibitory effect on CCCP-induced maximal OCR in permeabilized tumor cells or isolated brain mitochondria

**Fig 9:**
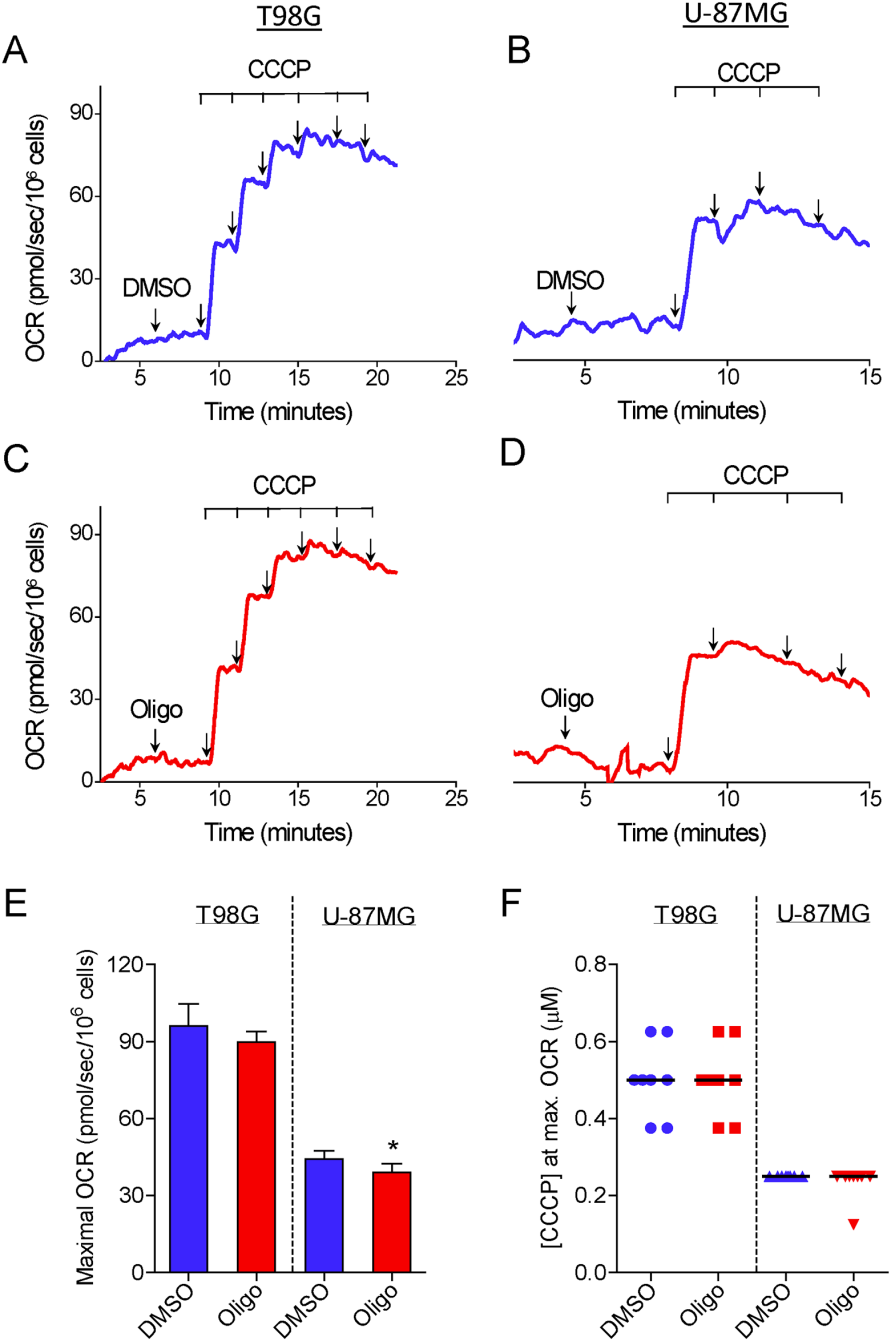
Effect of oligomycin on CCCP-induced maximal oxygen consumption in digitonin-permeabilized human glioma cells. A 125-μL aliquot of either T98G (3×10^6^ cells) or U-87MG (4×10^6^ cells) cell suspension was added to the final volume of 2 mL of reaction medium (125 mM sucrose, 65 mM KCl, 10 mM HEPES-K^+^ pH 7.2, 2 mM K_2_HPO_4_, 1 mM MgCl_2_, 1 mM EGTA and a cocktail of the respiratory substrates α-ketoglutarate, malate, glutamate and pyruvate, 5 mM of each) containing 30 μM digitonin. **A** and **C**: Representative OCR traces in suspended T98G cells. **B** and **D**: Representative OCR traces in suspended U-87MG cells. Where indicated by the arrows, 1 μg/mL oligomycin (Oligo) and 0.5 μL DMSO were added followed by sequential additions of CCCP (0.125 μM each). At the beginning of the measurements, it took 3–5 min to obtain a stable basal OCR. **E**: Values of CCCP-induced maximal OCR for T98G and U-87MG cells in the presence and absence of oligomycin. *Statistically significant difference from the results for the respective control (DMSO), *P*<0.05. **F**: Values of CCCP concentrations required for maximal OCR in T98G and U-87MG cells in the presence and absence of oligomycin.

Fig 9 shows the results of CCCP-induced maximal OCR estimated in digitonin-permeabilized cells incubated in a reaction medium supplemented with exogenous respiratory substrates. Basal OCR was lower in permeabilized cells than in intact cells. This is due to the dilution of endogenous ADP after plasma membrane permeabilization with the consequent cessation of oxidative phosphorylation. In fact, the addition of oligomycin to respiring cells under basal conditions had little or no effect on OCR (Fig 9C and 9D). Unlike the results for intact tumor cells, the results in this case showed only a small inhibitory effect (11.5 ± 3.5%) of oligomycin on CCCP-induced maximal OCR estimated for U-87MG cells and no significant inhibitory effect when this parameter was estimated for T98G cells (Fig 9E). Maximal OCR in digitonin-permeabilized cells was about 75% of that observed with intact cells. Median concentrations of CCCP required to reach maximal OCR did not differ for glioma cells treated with oligomycin or with DMSO (Fig 9F). The CCCP concentration required to reach maximal OCR in permeabilized cells (0.5 μM for T98G and 0.25 μM U-87MG) was approximately 10 times lower than that for intact cells, mainly because of the absence of FBS in the incubation medium for the former. No significant increase in maximal OCR in permeabilized cells was observed when the medium was supplemented with 5 μM cytochrome *c* (results not shown), indicating that the cells did not lose cytochrome *c* following treatment with digitonin.

**Fig 10:**
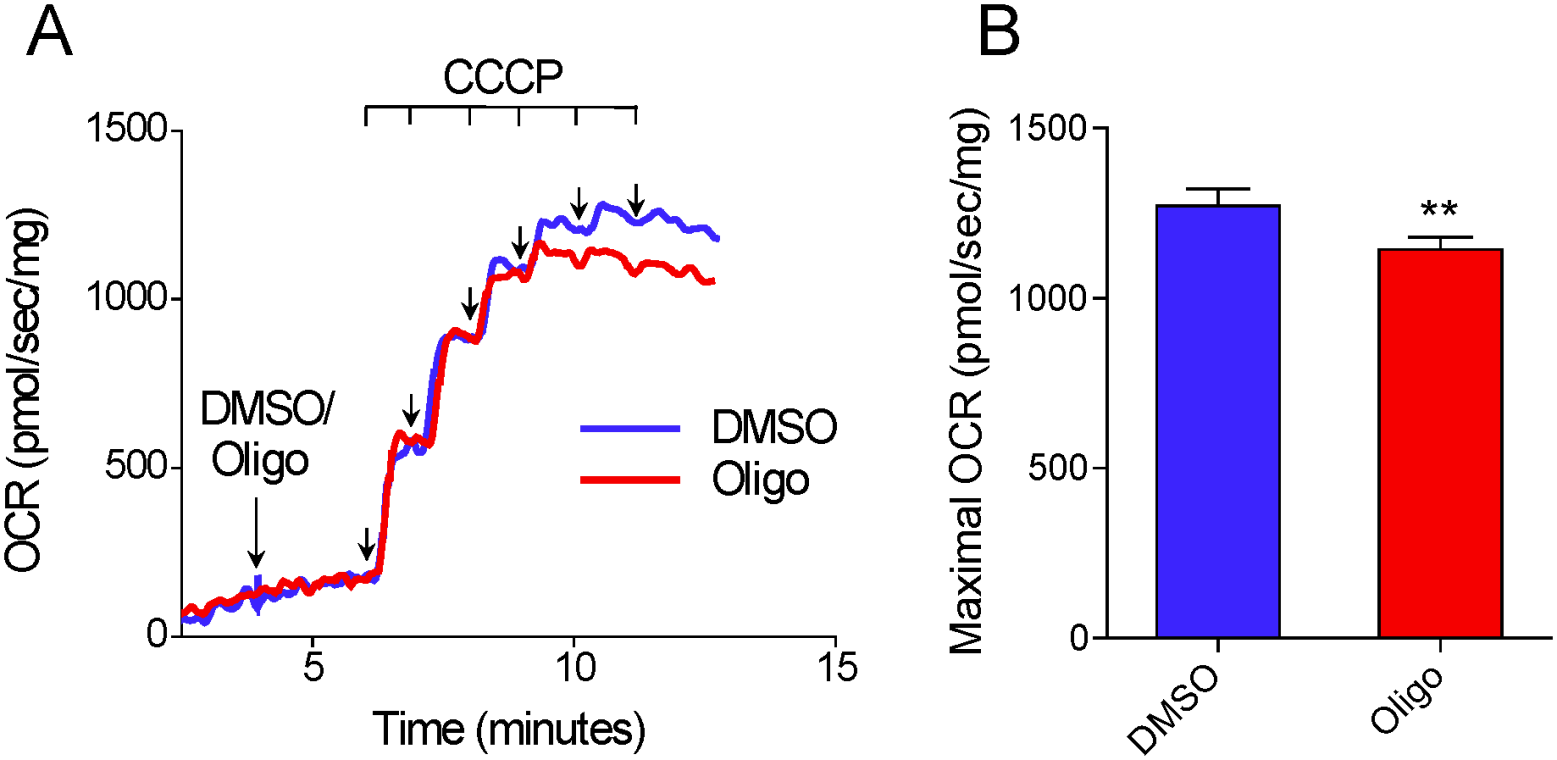
Oligomycin exerts only a minor inhibitory effect on CCCP-induced maximal oxygen consumption in isolated brain mitochondria. Isolated rat brain mitochondria (0.3 mg/mL) were incubated at 37°C in a 2 mL chamber containing 125 mM sucrose, 65 mM KCl, 10 mM HEPES-K^+^ pH 7.2, 2 mM K_2_HPO_4_, 1 mM MgCl_2_, and 1 mM EGTA. **A**: Representative traces of OCR in isolated brain mitochondria. Where indicated by the arrows, 1 μg/mL oligomycin (Oligo) or 0.5 μL DMSO were added, followed by sequential additions of CCCP (0.05 μM each). At the beginning of the measurements, it took 3–5 min to obtain a stable basal OCR. **B**: Values of CCCP-induced maximal OCR for brain mitochondria in the presence and absence of oligomycin. **Statistically significant difference from the results for DMSO, *P*<0.01.

As with permeabilized cells, CCCP-induced maximal OCR in isolated rat brain mitochondria was only slightly inhibited (10.0 ± 1.1%) when oligomycin was added before titration with CCCP (Fig 10).

## Discussion

The data reported here show that the presence of the ATP synthase inhibitors oligomycin or citreoviridin results in underestimation of CCCP- or FCCP-induced maximal OCR in intact cultured cells. This effect was observed in both attached (Fig 3) and suspended cells (Figs 1, 4 and 5) (S1–S3 Files), but was nearly abolished in digitonin-permeabilized cells (Fig 9). The lower maximal OCR in cells when ATP synthase inhibitors were present results in underestimation of the SRC. Under our experimental conditions, the SRC was underestimated by 25 to 45% when the tumor cell lines PC-3, T98G and U-87MG were assayed in the presence of oligomycin or citreoviridin. Oligomycin did not result in underestimation of SRC when maximal OCR was induced by the weaker protonophore DNP, but the lack of effect of oligomycin under these conditions is most likely related to the lower maximal OCR induced by DNP (Fig 7) as compared to more potent protonophores (i.e. CCCP or FCCP).

In the absence of ATP synthase inhibitors, higher concentrations of the protonophores CCCP and FCCP were required to achieve maximal OCR for intact cells (Figs 1 and 3–5) (S1 and S3 Files). This can be explained by the fact that when CCCP and FCCP promote a significant decrease in mitochondrial proton-motive force, reverse activity of ATP synthase occurs, with ATP hydrolysis driving proton pumping across the inner mitochondrial membrane [24, 33–35]. Under these conditions, the ATPase activity of ATP synthase occurs together with respiration-linked proton translocation through respiratory complexes I, III and IV into the intermembrane space. Thus, higher protonophore concentrations are required to dissipate the proton-motive force and promote maximal OCR. The ATP synthase inhibitors oligomycin [36] or citreoviridin (at a high concentration) [37] inhibit the forward and reverse activities of the ATP synthase. As discussed below, decreased respiratory chain activity may also contribute to the lower protonophore concentration required to achieve maximal OCR in the presence of ATP synthase inhibitors.

The inhibitory effect of oligomycin or citreoviridin on protonophore-induced maximal OCR seems to be associated with ATP synthase inhibition as the concentrations of these compounds required to achieve the reported undesired effect were similar to those that inhibit oxidative phosphorylation completely (Figs 2 and 5). ATP synthase inhibition may limit maximal OCR in cells by promoting changes in mitochondrial and cytosol adenylate energy charge, leading to decreased respiratory activity. Cells with high glycolytic activity, such as most tumor and proliferating non-tumor cells [3–5, 38], can partially maintain the intracellular ATP/ADP ratio even in the presence of respiratory chain inhibitors or protonophores as long as oligomycin is present to prevent consumption of ATP through reverse activity of ATP synthase [35, 38, 39]. A high intracellular ATP/ADP ratio may indirectly result in lower protonophore-induced maximal OCR because of a restraint in the availability or use of respiratory substrates by mitochondria. A high ATP/ADP ratio slows glycolysis [6, 40, 41], which generates the respiratory substrates glycerol-3-phosphate and pyruvate and limits the maximum activity of pyruvate dehydrogenase, glutamate dehydrogenase [42, 43] and the citric acid cycle enzymes citrate synthase and isocitrate dehydrogenase [44–46]. When cells are permeabilized or isolated mitochondria are used, a high concentration of exogenous respiratory substrate is provided and glycolysis is not present to promote ATP resynthesis. Under these conditions the inhibitory effect of oligomycin on CCCP-induced maximal OCR is minimized (Figs 9 and 10). In addition, ATP synthase inhibitors can also interact with non-specific targets, such as plasma membrane Na^+^/K^+^-ATPase and metabolic enzymes [47–49]. This may indirectly alter cellular energy metabolism and/or mitochondrial membrane conformation, limiting the maximal OCR induced by protonophores.

Interestingly, our results indicate that the ANT inhibitors BKA and CAT had no effect on oxidative phosphorylation in intact T98G cells (Fig 6A and 6C) and therefore preclude the use of these compounds to substitute ATP synthase inhibitors in the protocol for estimation of maximal OCR in cells with previously inhibited oxidative phosphorylation. Nonetheless, BKA and CAT were effective inhibitors of oxidative phosphorylation in digitonin-permeabilized cells (Fig 6B and 6D). The fact that BKA and CAT had no effect on intact tumor cells may be due to the poor permeability of these compounds through the plasma membrane of tumor cells, the effective removal of these compounds from tumor cells via multidrug resistant ATP-binding cassette (ABC) transporters [50], or, as recently proposed by Maldonado and Lemasters [6], a different mechanism of mitochondrial ADP/ATP transport in tumor cells, possibly involving the ATP-Mg/P_i_ carrier. In fact, the ATP-Mg/P_i_ carrier is overexpressed in many tumor cells [51]. In the case of this last hypothesis, why the mitochondrial ATP-Mg/P_i_ carrier does not transport ADP/ATP in digitonin-permeabilized T98G cells (Fig 6B and 6D) remains to be clarified.

## Conclusion

Maximal OCR and SRC are valuable parameters that are widely used to assess cellular respiratory capacity in normal and tumor cells. Therefore, the effects of ATP synthase inhibitors on CCCP- or FCCP-induced maximal OCR and, consequently, on the estimation of SRC, should be carefully considered to avoid flawed interpretations. We conclude that unless a previously validated protocol is used, maximal OCR in intact cells should be determined in the absence of ATP synthase inhibitors.

## Supporting Information

S1 File Inhibitory effect of oligomycin on CCCP-induced maximal oxygen consumption in PC3 cells. **A** and **B**: Representative OCR traces in suspended PC3 cells (1.5×10^6^ cells/mL). Where indicated by the arrows, 1 μg/mL oligomycin (Oligo) or 0.5 μL DMSO were added, followed by sequential additions of CCCP (1 μM each). **C**: SRC values for PC3 cells in the presence and absence of oligomycin. **Statistically significant difference from the results for DMSO, *P*<0.01. **D**: Values of CCCP concentrations required to achieve maximal oxygen consumption rate in PC3 cells in the presence and absence of oligomycin.
(PDF)

S2 File Inhibitory effect of oligomycin on CCCP-stimulated oxygen consumption by T98G cells using a single addition of CCCP. T98G cells (1.5×10^6^ cells/mL) were incubated and where indicated by the arrows, 0.5 μL DMSO or 1 μg/mL oligomycin (Oligo) followed by CCCP were added (**A**: sequential additions of 1 μM CCCP; **B** and **C**: only one addition of 6 μM CCCP). Results are shown as percentages of OCR determined just before the addition of DMSO or oligomycin.
(PDF)

S3 File Inhibitory effect of oligomycin on FCCP-induced maximal oxygen consumption by T98G cells. **A** and **B**: Representative OCR traces by suspended T98G cells (1.5×10^6^ cells/mL). Where indicated by the arrows, 1 μg/mL oligomycin (Oligo) or 0.5 μL DMSO were added followed by sequential additions of FCCP (1 μM each). **C**: SRC values for T98G cells in the presence and absence of oligomycin. **Statistically significant difference from the results for DMSO, *P*<0.01. **D**: FCCP concentrations required for maximal OCR in T98G cells in the presence and absence of oligomycin.
(PDF)
