# Supplementary figures and images for: Underestimation of the Maximal Capacity of the Mitochondrial Electron Transport System in Oligomycin-Treated Cells
Source: PLoS One. 2016 Mar 7;11(3):e0150967. doi: 10.1371/journal.pone.0150967 (PMC4780810; doi:10.1371/journal.pone.0150967)

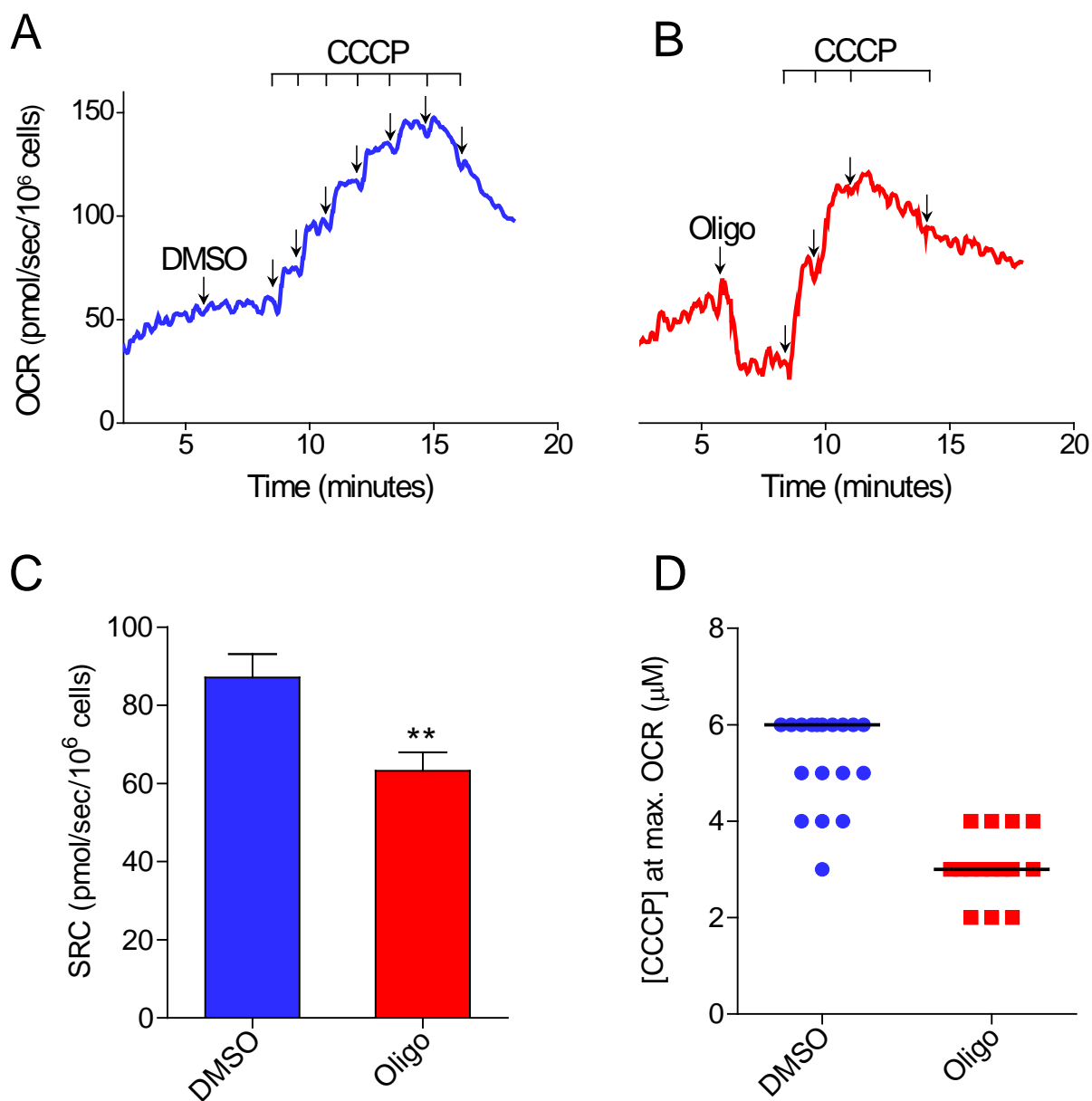

**S1 Fig.**

**Inhibitory effect of oligomycin on CCCP-induced maximal oxygen consumption in PC3 cells.**

Supplement: S1 File — A and B: Representative OCR traces in suspended PC3 cells (1.5×10^6^ cells/mL). Where indicated by the arrows, 1 μg/mL oligomycin (Oligo) or 0.5 μL DMSO were added, followed by sequential additions of CCCP (1 μM each). C: SRC values for PC3 cells in the presence and absence of oligomycin. **Statistically significant difference from the results for DMSO, P<0.01. D: Values of CCCP concentrations required to achieve maximal oxygen consumption rate in PC3 cells in the presence and absence of oligomycin. (PDF) [file pone.0150967.s001.pdf]

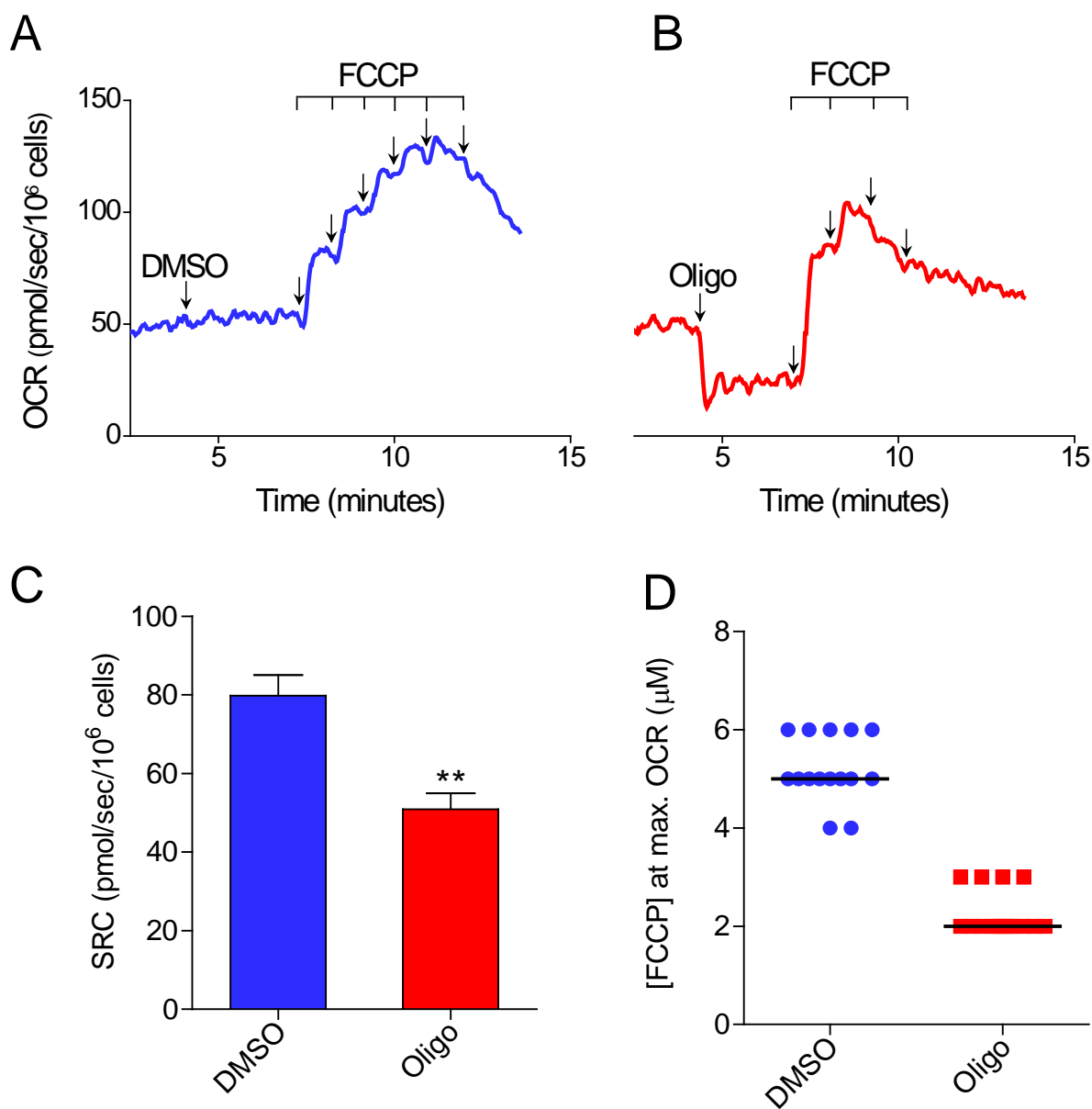

**S3 Fig.**

**Inhibitory effect of oligomycin on FCCP-induced maximal oxygen consumption by T98G cells.**

Supplement: S3 File — A and B: Representative OCR traces by suspended T98G cells (1.5×10^6^ cells/mL). Where indicated by the arrows, 1 μg/mL oligomycin (Oligo) or 0.5 μL DMSO were added followed by sequential additions of FCCP (1 μM each). C: SRC values for T98G cells in the presence and absence of oligomycin. **Statistically significant difference from the results for DMSO, P<0.01. D: FCCP concentrations required for maximal OCR in T98G cells in the presence and absence of oligomycin. (PDF) [file pone.0150967.s003.pdf]
