# Supplementary material for: Underestimation of the Maximal Capacity of the Mitochondrial Electron Transport System in Oligomycin-Treated Cells
Source: PLoS One. 2016 Mar 7;11(3):e0150967. doi: 10.1371/journal.pone.0150967 (PMC4780810; doi:10.1371/journal.pone.0150967)
Supplement: S2 File — T98G cells (1.5×10^6^ cells/mL) were incubated and where indicated by the arrows, 0.5 μL DMSO or 1 μg/mL oligomycin (Oligo) followed by CCCP were added (A: sequential additions of 1 μM CCCP; B and C: only one addition of 6 μM CCCP). Results are shown as percentages of OCR determined just before the addition of DMSO or oligomycin. (PDF) [file pone.0150967.s002.pdf]

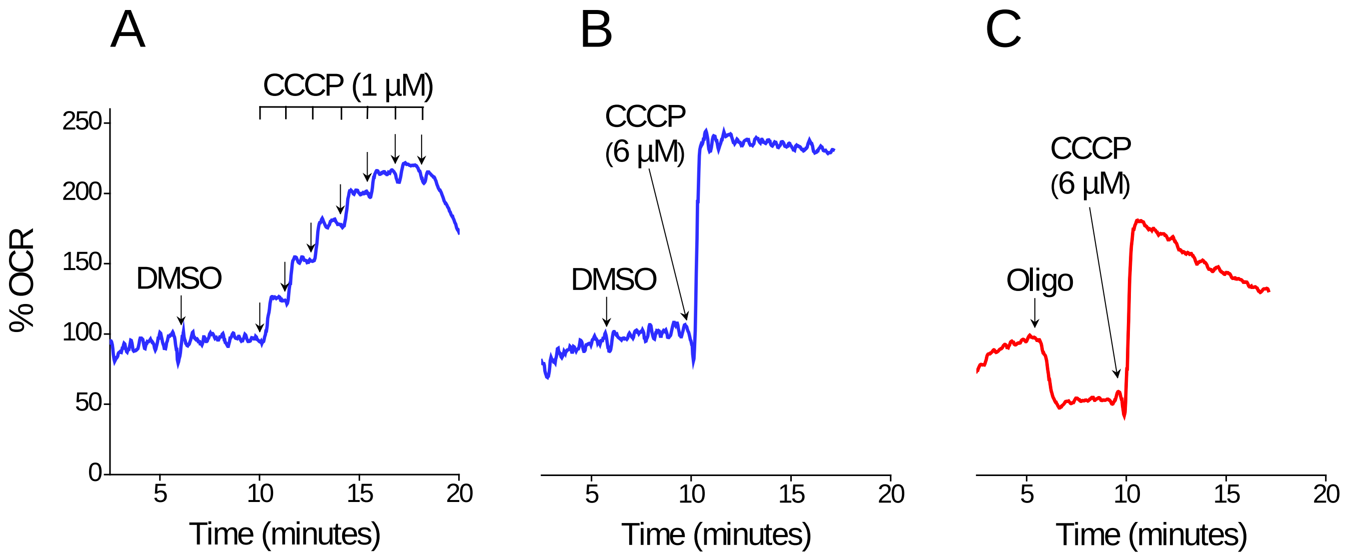

S2 Fig.

Inhibitory effect of oligomycin on CCCP-stimulated oxygen consumption by T98G cells using a single addition of CCCP.
